# Reply: Diagnostic accuracy of ChatGPT-4 and liver fibrosis in MASH

**DOI:** 10.1097/HC9.0000000000000764

**Published:** 2025-07-21

**Authors:** Davide Panzeri, Luca Di Tommaso, Laura Sironi

**Affiliations:** 1 Leibniz-Institut für Analytische Wissenschaften - ISAS - e.V., Dortmund, Germany; 2 Department of Biomedical Sciences, Humanitas University, Milan, Italy; 3 Department of Pathology, IRCCS Humanitas Research Hospital, Milan, Italy; 4 Department of Physics, University of Milano-Bicocca, Milan, Italy

To the editor,

We welcome this opportunity to further clarify our methodology and findings. The correspondents raised several points we would like to address.

Regarding the sample size, there seems to be a potential misunderstanding. To assess ChatGPT-4-vision’s diagnostic accuracy, we exploited 59 whole slide images (WSIs) from 59 different patients with metabolic dysfunction–associated steatohepatitis (N=59), not merely 59 “photos”. For the expert-selected data set derived from the 59 WSIs, an external pathologist extracted at least 5 fields of view (FOVs) at ×4 magnification, 10 FOVs at ×10, and 20 FOVs at ×20 magnification per WSI.^1^ Therefore, the model’s assessment was based on the evaluation of at least 400 FOVs. We agree, as noted in our discussion, that larger, multicentric studies are essential for validating performance across broader populations. Our data set, however, provided a robust basis for the initial comparative evaluation presented.

We already addressed the potential influence of FOV selection in our manuscript.^1^ Our methodology explicitly included a comparison between FOVs selected by an expert liver pathologist and randomly cropped FOVs to assess the impact of user expertise and potential selection bias. Our strategy of selecting multiple FOVs at different magnifications from various locations across the WSI, repeated over 3 distinct conversation sessions, was specifically designed to account for the heterogeneity inherent in liver biopsies and ensure the model was exposed to diverse representations of the tissue. Furthermore, the cases selected met specific inclusion criteria and were sourced from a private data set, ensuring no prior model exposure.^1^

Our study employed state-of-the-art methods for evaluating diagnostic accuracy and interobserver agreement, including confusion matrices, overall accuracy, recall (sensitivity) per stage, Cohen Kappa with quadratic weighting, and Fisher exact test for comparisons. We believe the statistical analyses performed were appropriate for the study’s objectives.

Our study was specifically designed to evaluate ChatGPT-4’s performance on the histopathological interpretation task of fibrosis staging from images, mirroring how pathologists initially assess slides. As stated in our discussion,^1^ large language models are potential supportive tools, and their integration into clinical practice requires careful consideration of broader context, limitations, and ethical aspects.

Regarding in-context learning, the observed improvement in accuracy should not be confused with true model generalization in the traditional machine learning sense (which typically involves retraining or fine-tuning on diverse data sets). Our study showed that ChatGPT-4 could adapt its interpretation and learn to recognize specific features when appropriately prompted with external examples.﻿ This demonstrates learning within the provided context, rather than simple image recognition.^1^

Finally, it is likely that a Convolutional Neural Network specifically trained for fibrosis staging could achieve similar, if not better, performance. However, developing and deploying such specialized models presents its own challenges. Furthermore, Convolutional Neural Network implementation often requires dedicated infrastructure, and they generally lack the interactive, conversational interface offered by large language models like ChatGPT. Large language models offer the advantage of being pretrained and accessible via a web interface, potentially lowering the barrier for pathologists to explore AI assistance.

In conclusion, our study provides valuable initial data on ChatGPT-4’s capabilities in metabolic dysfunction–associated steatohepatitis fibrosis staging, demonstrating accuracy comparable to expert pathologists, particularly when guided by in-context learning.
